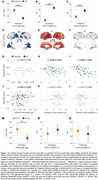# Distinct associations of excitatory‐inhibitory (E/I) imbalance in aging and in Alzheimer’s disease

**DOI:** 10.1002/alz.091606

**Published:** 2025-01-03

**Authors:** Kamalini G Ranasinghe, Parul Verma, Kiwamu Kudo, Faatimah Syed, Katherine P. Rankin, Bruce L. Miller, Joel H. Kramer, Gil D. Rabinovici, Heidi Kirsch, Keith Vossel, Ashish Raj, Srikantan S Nagarajan

**Affiliations:** ^1^ University of California San Francisco, San Francisco, CA USA; ^2^ Medical Imaging Business Center, Ricoh Company, Kanazawa Japan; ^3^ Memory & Aging Center, Department of Neurology, University of California in San Francisco, San Francisco, CA USA; ^4^ Memory and Aging Center, Weill Institute for Neurosciences, University of California, San Francisco (UCSF), San Francisco, CA USA; ^5^ University of California Los Angeles, Los Angeles, CA USA

## Abstract

**Background:**

Neural circuit hyperexcitability and impaired excitation‐to‐inhibition (E/I) activity is believed to be a key contributor to synaptic and network degeneration in Alzheimer’s disease (AD). Extensive preclinical research on transgenic animal models of AD have demonstrated neuronal and circuit level E/I imbalance mediated by amyloid‐beta (Aβ) and tau proteins. Synaptic and network deficits are also integral changes of aging. It is unknown whether the mechanisms of E/I imbalance in aging are distinct from that in AD. In this study we leveraged the aperiodic spectral slope measure, which represent the spontaneous neuronal firing, and which has been shown to indicate higher E/I as reduced slope values.

**Methods:**

We used source reconstructed, high spatiotemporal resolution signals, from magnetoencephalography (MEG) for the spectral analysis and used the neural mass model (NMM) to estimate excitatory and inhibitory neuronal parameters, in patients with AD (n = 85) and age‐matched elderly‐controls (n = 45). The NMM estimates the time constants of excitatory (t_e_) and inhibitory (t_i_) neuronal subpopulations and depicts E/I as 1/inhibitory‐neural‐gain (g_ii_). We estimated the aperiodic slope from the spectral analysis (15‐50 Hz) and examined the correlations with NMM parameters. In a subset of AD patients, we recorded the presence or absence of epileptiform activity (EPI) and examined the associations of neuronal‐time‐contents and inhibitory‐neural‐gain in AD‐EPI+ (n = 20) vs. AD‐EPI− (n = 30).

**Results:**

AD patients showed reduced inhibitory‐neural‐gains compared to elderly‐controls indicating increased E/I (Fig. 1.A). AD patients also showed increased neuronal‐time‐constants than controls (Fig. 1.B‐C). The spatial patterns of reduced inhibitory‐neural‐gains overlapped with AD vulnerable anatomic regions showing a posterior temporo‐parietal‐occipital distribution (Fig. 1.D). Greater reductions of aperiodic‐slope distinctly correlated with inhibitory‐neural‐gains in AD and with higher neuronal‐time‐constants in elderly‐controls (Fig. 1.G‐L). Moreover, AD‐EPI+ showed greater reductions in inhibitory‐neural‐gains than AD‐EPI−, but no differences in neuronal‐time‐constants (Fig. 1.M‐O).

**Conclusions:**

Our findings identified impaired inhibitory synaptic activity as the strongest correlate of neural circuit hyperexcitability in AD. In contrast, impaired timing of both excitatory and inhibitory neurons were the best predictors of neural circuit hyperexcitability in aging. These results suggest different mechanisms of neural circuit hyperexcitability in AD vs. aging and have important implications to translational as well as clinical studies probing early network changes.